# Ultrasonography and CT examination of ovarian follicular development in ‘*Testudo graeca*’ during 1 year in captivity

**DOI:** 10.1002/vms3.1259

**Published:** 2023-09-05

**Authors:** Banafsheh Shateri Amiri, Sarang Soroori, Amir Rostami, Mohammad Molazem, Alireza Bahonar

**Affiliations:** ^1^ Department of Surgery and Radiology Faculty of Veterinary Medicine University of Tehran Tehran Iran; ^2^ Department of Internal Medicine Faculty of Veterinary Medicine University of Tehran Tehran Iran; ^3^ Department of Food Hygiene and Quality Faculty of Veterinary Medicine University of Tehran Tehran Iran

**Keywords:** captivity, CT examination, ovarian follicle, *Testudo Graeca*, ultrasonography

## Abstract

**Background:**

The Greek or Mediterranean tortoise (*Testudo graeca*), commonly known as the spur‐thighed tortoise, is a species of tortoise in the family *Testudinidae* that is found in North Africa, Western Asia and Europe. Several species of this genus are under threat in the wild, mainly from habitat destruction therefore, accurate information about the sexual cycle and the exact time of follicular development can be effective in successful reproduction planning and preventing species extinction.

**Objective:**

The experimental part of this study is focused on the evaluation of the reproductive apparatus of tortoises and development of follicles in ovaries during 1 year by ultrasonography and computed tomography.

**Methods:**

Ultrasonography and CT scanning was carried out on 10 female ‘*T. Graeca*’ tortoises which were maintained at Tehran Eram Zoo from November 2021 to November 2022 (1 year). Ultrasonography and CT exams were performed on all animals.

**Results:**

In the first study, on 28 November 2021, the ovarian cycle was characterized by the presence of previtellogenic follicles and atretic follicles. The numbers of follicles were best demonstrated on CT examination. Ultrasonography and CT scan in the second study showed no change in type of follicles from previous study. In the third study, atretic follicles were more than previtellogenic follicles, whereas in the fourth one, the numbers of previtellogenic follicles were more than atretic follicles. In following, at 6 and 29 June 2022, the absence of atretic follicles and just presence of previtellogenic follicles were observed. On 20 July 2022, the presence of a few number of atretic follicles and more previtellogenic follicles were noticed. On 30 July 2022, the absence of atretic follicles and presence of previtellogenic follicles and, for the first time, preovulatory follicles were obvious. In four following studies the absence of atretic follicles and the presence of previtellogenic follicles were diagnosed. In the last study, the absence of atretic follicles and the presence of preovulatory and previtellogenic follicles were observed.

**Conclusion:**

In current study, the reproductive cycle of female *T. Graeca* is evaluated during 1 year in captivity with ultrasonography and CT scan. It can be concluded that in captivity, the reproductive cycle does not result in the formation of eggs or calcareous shells and atretic follicles in the study year continued as before; so this should be highly considered in captivity breeding programmes. This study also revealed that among imaging modalities, CT scan is the best modality for detecting the shape, size, type and numbers of the follicles for further evaluation of location and shape of the follicles.

## Graphical abstract

Review of ultrasonography and CT images of ovarian follicles, data analysis and ultrasonography approach.

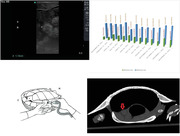

## 1 INTRODUCTION

The Greek or Mediterranean tortoise (*Testudo graeca*), commonly known as the spur‐thighed tortoise, is a species of tortoise in the family *Testudinidae* that found is in North Africa, Western Asia and Europe. *T. graeca graeca* is relatively small: 13–16 cm. This species can measure up to 30 cm in carapace length with a maximum weight of approximately 6 kg (Vlachos & Tsoukala, 2014). The Greek tortoise is a very long‐lived animal, achieving a lifespan upwards of 125 years, with some unverified reports up to 200 years (Nijman & Bergin, 2017). In *T. graeca*, immediately after waking from hibernation, the mating instinct starts up (Nijman & Bergin, 2017).

Several species of this genus are under threat in the wild, mainly from habitat destruction (Vlachos & Tsoukala, 2014); therefore, accurate information about the sexual cycle and the exact time of follicular development can be effective in successful planning of reproduction and preventing the extinction of this species.

For example, some species of reptile have been shown to commence vitellogenesis in the late summer/fall but to complete gamete production (preovulatory follicles) only in spring of the following year (Blanvillain et al., 2011).

Most tortoises appear capable of annual breeding when in appropriate habitats and under ideal environmental conditions. In particular, most tortoises seem to breed annually. Interestingly, some adult females may not reproduce in a given year. The desert tortoise exhibits mating activity twice a year (in the spring and in the fall) (Blanvillain et al., 2011).

In the past, hormonal cycles of reptiles were explicated by using direct methods to evaluate plasma hormone levels by RIA (radioimmunoassay) or more recently enzyme‐linked immunosorbent assay. This has provided an opportunity to those interested in studying the regulatory physiology of reptiles. On the other hand, finding tortoises can be very hard at times, especially during hibernation when they are understandably less well studied.

Endoscopy and laparoscopy are the other diagnostic methods which are more invasive techniques involving a surgical procedure allowing one to look directly at the gonads and therefore to obtain information on gender, maturity status and reproductive state of the tortoise through direct gonadal visualization and tissue biopsy (Blanvillain et al., 2011).

Among diagnostic imaging modalities, radiography is a useful technique to determine shelled eggs; however; this technique does not allow the visualization of other structures such as ovarian follicles.

Ultrasonography can replace the techniques described above in many instances, as it allows researchers to visualize and measure follicles and eggs. In normal and optimal instances in wild, follicles had reached ovulatory size prior to hibernation, and shelled eggs were observed in the oviducts in mid‐April (Blanvillain et al., 2011).

Ultrasonography provides a more complete and accurate assessment of reproductive status without being limited to the detection of only calcified eggs via radiography, or only determining gravid/non‐gravid status via palpation (Donini et al., 2017).

Even though ultrasonography provided a better reproductive assessment, a complete picture is often unobtainable. Ultrasounds generally are only able to predict a minimum clutch size due to the location of the oviducts in chelonians (Donini et al., 2017). Some eggs situated in the anterior portion of the oviducts will go undetected by the probe. It is unlikely that all follicles observed will be ovulated and become eggs, as follicles often become atretic near the end of the reproductive season (Donini et al., 2017). Thus, follicles identified in early portions of a reproductive cycle may not culminate in eggs before being broken down during atresia (Donini et al., 2017).

In ultrasonography, the ovaries are visible depending on their size and phase of the reproductive cycle. At the beginning of this cycle, the ovarian follicles are anechoic. Their size varies from 1 mm up to several mm. Before ovulation, they grow; appear as large circular structures and their echogenicity increases. In the late pre‐ovulation phase, almost the entire abdominal cavity is filled up with round follicles. After ovulation, the eggs grow and the wall turns hyperechoic (Urbanová & Halán, 2016).

CT is being increasingly utilized for diagnostic imaging evaluation procedures for reptile patients due to increased availability for veterinary practitioners.^1^ In a CT examination, sectional images are produced with a three‐dimensional method. Using reformation and the respective reconstructions, various planes and also 3D models can be produced and assessed.^1^ The length of the investigation usually lasts for a maximum of approximately 90 s, which is very short in comparison to other imaging modalities such as MRI (Silverman, 1993). Another reason why CT is superior to MRI is the lack of necessity of anaesthesia. The scanners used are usually those designed for human medicine; however, the examination parameters must be applied to the patient (Silverman, 1993).

## 2 METHODS AND MATERIALS

The experimental part of this study is focused on the examination of the reproductive apparatus of tortoises and evaluation of development of follicles in ovaries during 1 year.

Ultrasonography and CT scanning were carried out on 10 female ‘*T. graeca*’ tortoises which were maintained at Tehran Eram Zoo from November 2021 to November 2022 (1 year). Morphologic and morphometric evaluation of follicles in ultrasonography and CT exams were performed with all animals. Only clinically healthy animals were included in the study. Descriptive data analysis was performed by calculating the mean ± SD and the maximum/minimum size of follicles in each time of ultrasonography and computed tomographic examination, using Excel software.

### 2.1 Ultrasonographic exam

**FIGURE 1:**
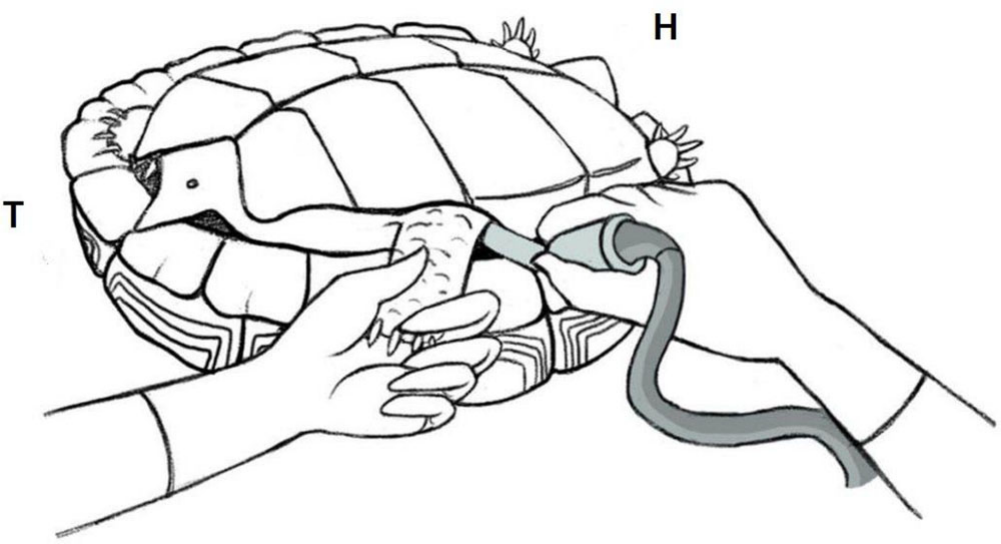
Schematic image of ultrasonography approach. H, head; T, tail.

The ultrasonographic procedure was accomplished with the animal in dorsal decumbency, and the hindlimb, ipsilateral to the ovary, extended and properly immobilized (Figure 1). Then, transducer hockey stick probe was enough to access organs of the coelomic cavity in the species studied. A coupling gel was placed against the soft tissue of the inguinal region, and the scanning was performed orienting the probe in a craniolateral and craniomedial directions in prefemoral window. No sedation was required for the examination. In ultrasonography, the types, size and echogenicity of follicles were evaluated in each study and each animal.

### 2.2 CT examinations

For CT examination, animals were placed in ventral recumbency and in a perspex box to be fixed and prevent motion unsharpness artefacts during scanning. Images were evaluated in transverse plane with WW: 1500, WL: 450. Technical factors were kV: 130, mAs: 97, slice thickness: 1 mm, pitch: 0.95, rotation time: 0.8 s. No sedation was required for the examination. In CT, the types, numbers, size and the Hounsfield unit of follicles were investigated.

### 2.3 Data assessments

Ultrasonography and computed tomographic examinations were done by a board‐certified veterinary radiologist under the supervision of an associate professor of radiology department, an associate professor of small animal internal medicine department and an assistant professor with board certification of European college of veterinary diagnostic imaging.

## 3 RESULTS

### 3.1 Ultrasonographic examination

From 28 November 2021 to 22 November 2022, the tortoises were scanned 13 times by ultrasonography to explore the ovarian cycle.

### 3.2 Types of follicles

**FIGURE 2:**
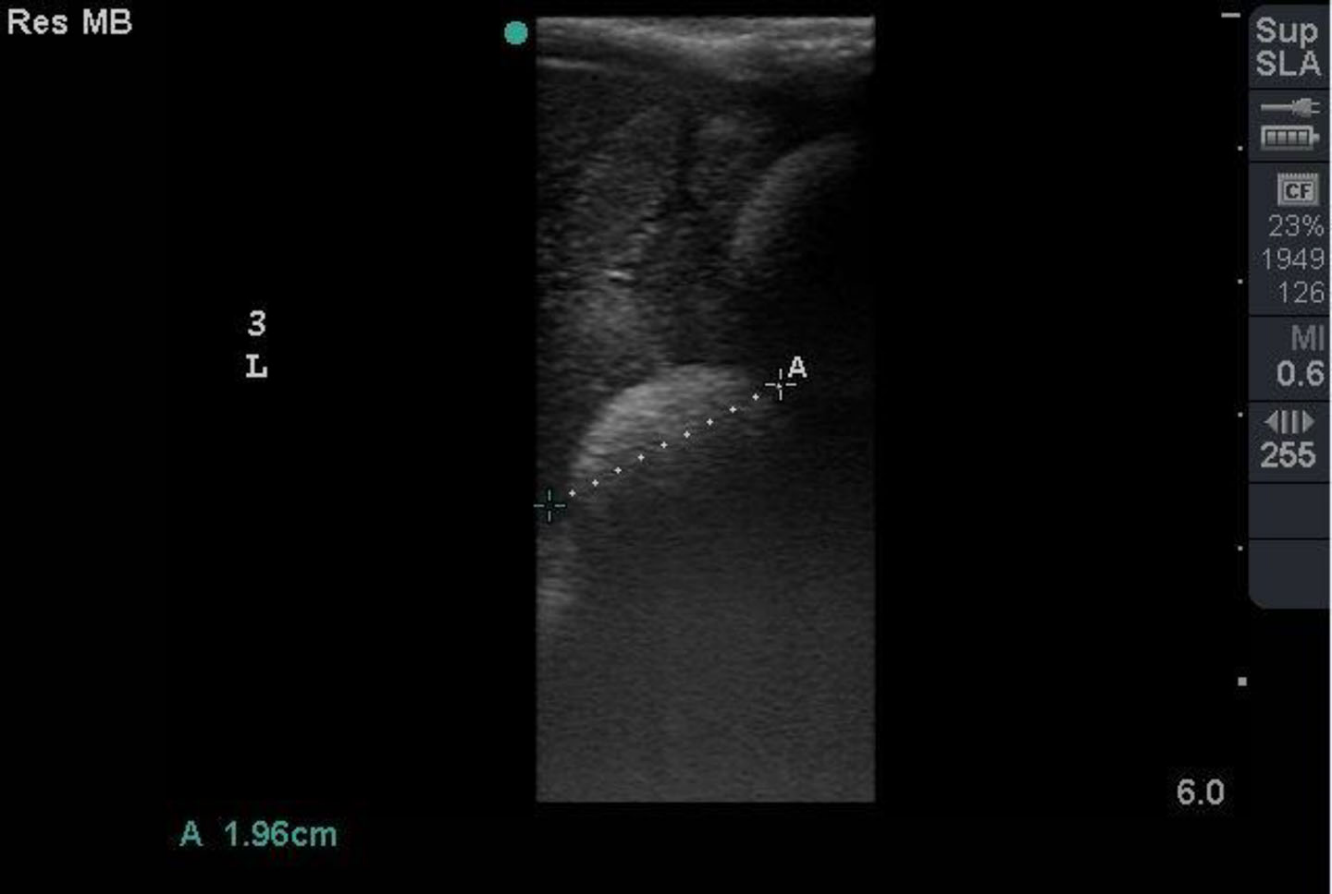
Previtellogenic follicle with hyperechoic surface with distal acoustic shadowing artefact.

**FIGURE 3:**
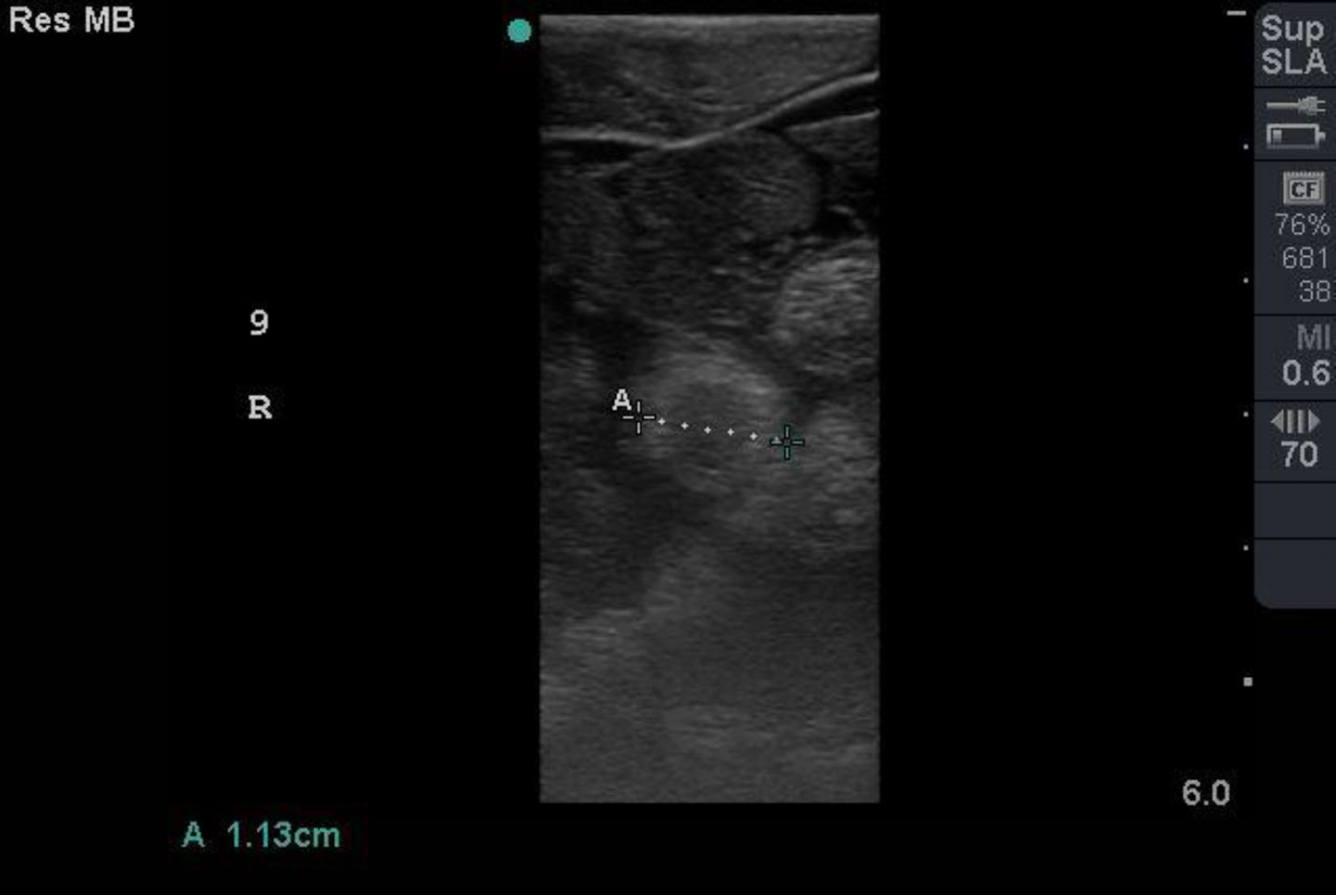
Atretic follicle, smallest follicle with hypoechoic centre and hyperechoic rim with no significant distal acoustic shadowing artefact.

**FIGURE 4:**
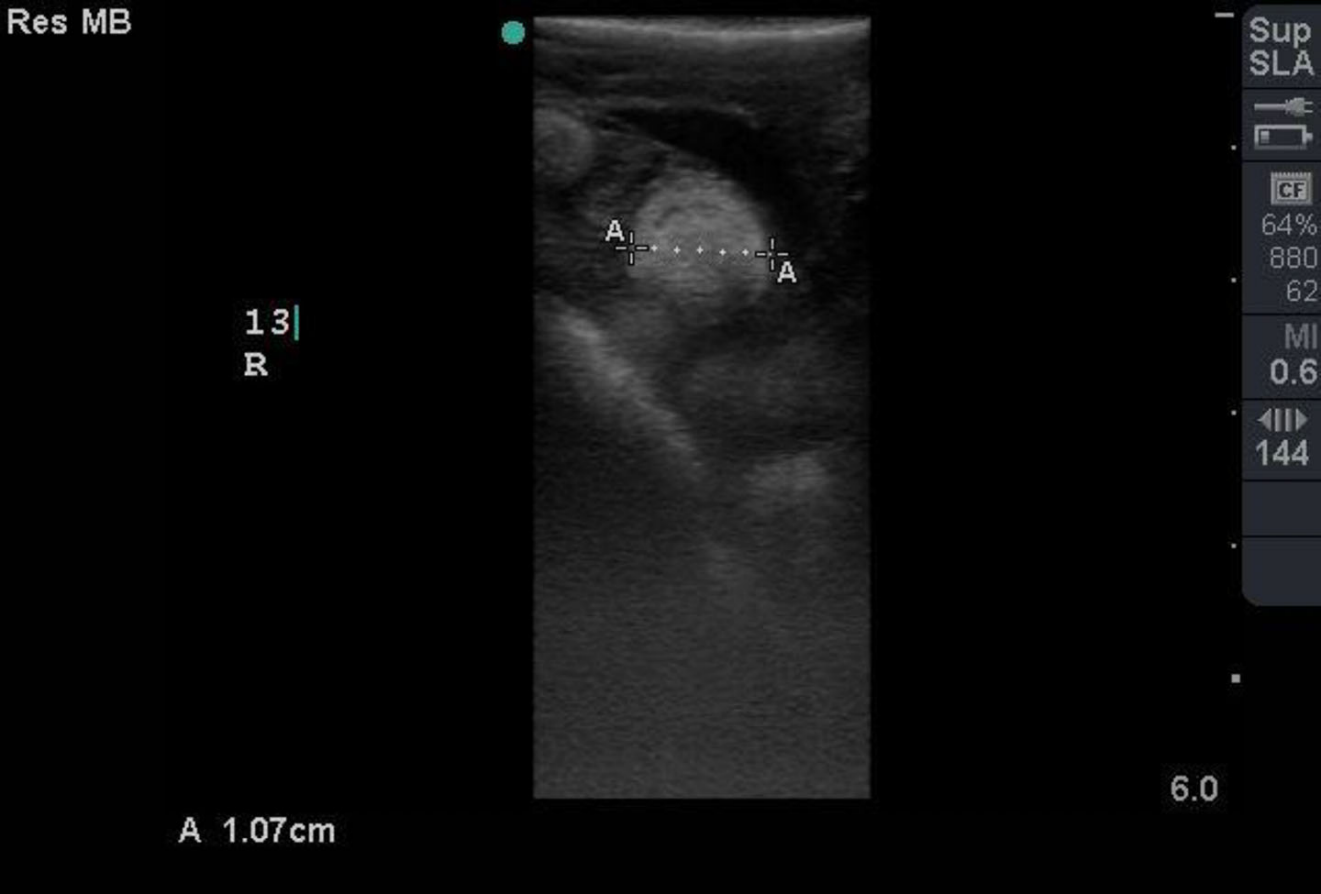
Preovulatory follicle with the most echogenicity in centre and peripheral with no significant distal acoustic shadowing artefact.

In the first study on 28 November 2021, the ovarian cycle was characterized by the presence of previtellogenic follicles (hyperechoic surface with dirt distal acoustic shadowing artefact) (Figure 2) and atretic follicles (smallest follicle with hypoechoic centre and hyperechoic rim with no significant distal acoustic shadowing artefact) (Figure 3). The numbers of follicles were best demonstrated on CT examination. Ultrasonographic scanning on 27 February 2021 showed no change of follicles type from previous study. On 18 April 2022, atretic follicles were more than previtellogenic follicles, whereas on 18 May 2022, the numbers of previtellogenic follicles were more than atretic follicles. In following, on 6 and 29 June 2022, the absence of atretic follicles and just presence of previtellogenic follicles were observed. On 20 July 2022, presence of a few number of atretic follicles and more previtellogenic follicles were noticed. On 30 July 2022, the absence of atretic follicles and the presence of previtellogenic follicles and, for the first time, preovulatory follicles (follicles with the most echogenicity in centre and peripheral with no significant distal acoustic shadowing artefact) (Figure 4) were obvious. On 10 August until 1 November 2022 (4 studies), the absence of atretic follicles and the presence of previtellogenic follicles were diagnosed. In the last study on 22 November 2022, the absence of atretic follicles and the presence of preovulatory and previtellogenic follicles were observed.

### 3.3 Size of follicles

**FIGURE 5:**
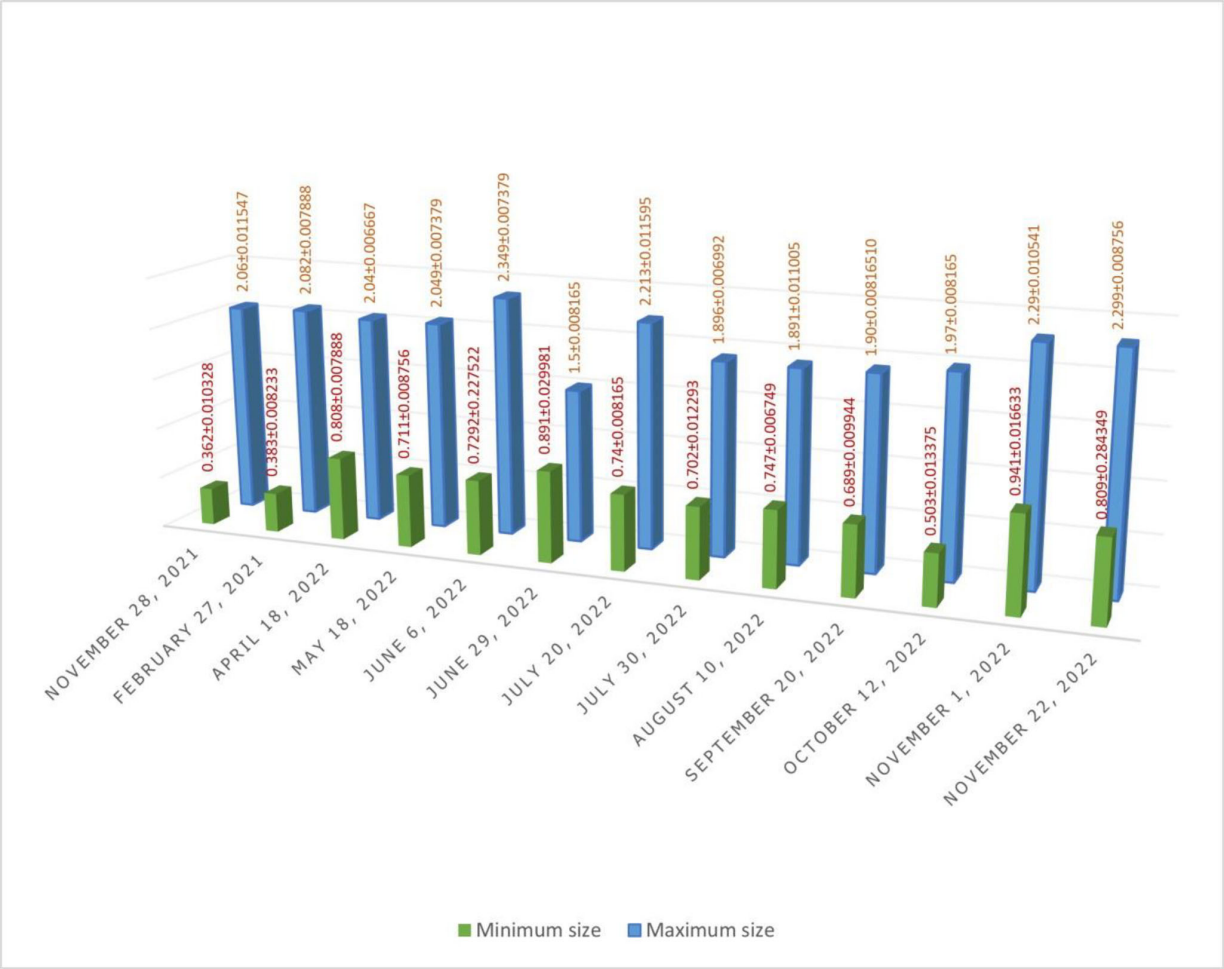
The column chart of maximum and minimum sizes (mean ± SD) of follicles in ultrasonography examination of 10 tortoises (cm).

The maximum and minimum sizes of follicles are explained in Table 1. From the beginning of the study on 28 November until 18 May, no meaningful difference was observed in follicular size with increase in size on 6 June. Meaningful decrease in follicular size on 20 July and the following increased size was observed on 30 July. No meaningful difference was observed in four following studies until 22 November 2022, when follicular size was observed to increase. Sizes of follicles in different times are shown in Table 1 and the column chart of Figure 5.

**TABLE 1:** The maximum and minimum size (mean ± SD) of follicles in ultrasonographic examination of 10 tortoises (cm).

Minimum size (mean ± SD)	Maximum size (mean ± SD)	Date of study
0.362 ± 0.010328	2.06 ± 0.011547	28 November 2021
0.383 ± 0.008233	2.082 ± 0.007888	27 February 2021
0.808 ± 0.007888	2.04 ± 0.006667	18 April 2022
0.711 ± 0.008756	2.049 ± 0.007379	18 May 2022
0.7292 ± 0.227522	2.349 ± 0.007379	6 June 2022
0.891 ± 0.029981	1.5 ± 0.008165	29 June 2022
0.74 ± 0.008165	2.213 ± 0.011595	20 July 2022
0.702 ± 0.012293	1.896 ± 0.006992	30 July 2022
0.747 ± 0.006749	1.891 ± 0.011005	10 august 2022
0.689 ± 0.009944	1.90 ± 0.00816510	20 September 2022
0.503 ± 0.013375	1.97 ± 0.008165	12 October 2022
0.941 ± 0.016633	2.29 ± 0.010541	1 November 2022
0.809 ± 0.284349	2.299 ± 0.008756	22 November 2022

### 3.4 Echogenicity

**FIGURE 6:**
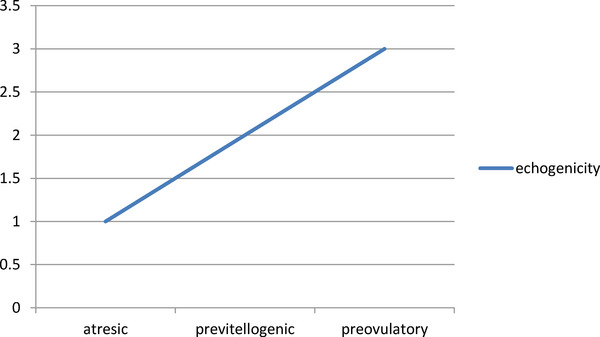
Quantification of follicular echogenicity compared to size of follicles in ultrasonography examination.

As the size of the follicles increased in this study, increased echogenicity of follicles was evident. Preovulatory follicles had the most echogenicity rather than previtellogenic and atretic follicles, respectively (Figure 6). In order to better understand the relative echogenicity of the follicles in this study, we have quantified them as follows: preovulatory follicles (3), previtellogenic follicles (2) and atretic follicles (1).

### 3.5 CT examination

CT examinations were performed 13 times from 28 November 2021 until 22 November 2022 with the purpose of detecting follicles, attenuation, HU and size of follicles.

### 3.6 Types of follicles

**FIGURE 7:**
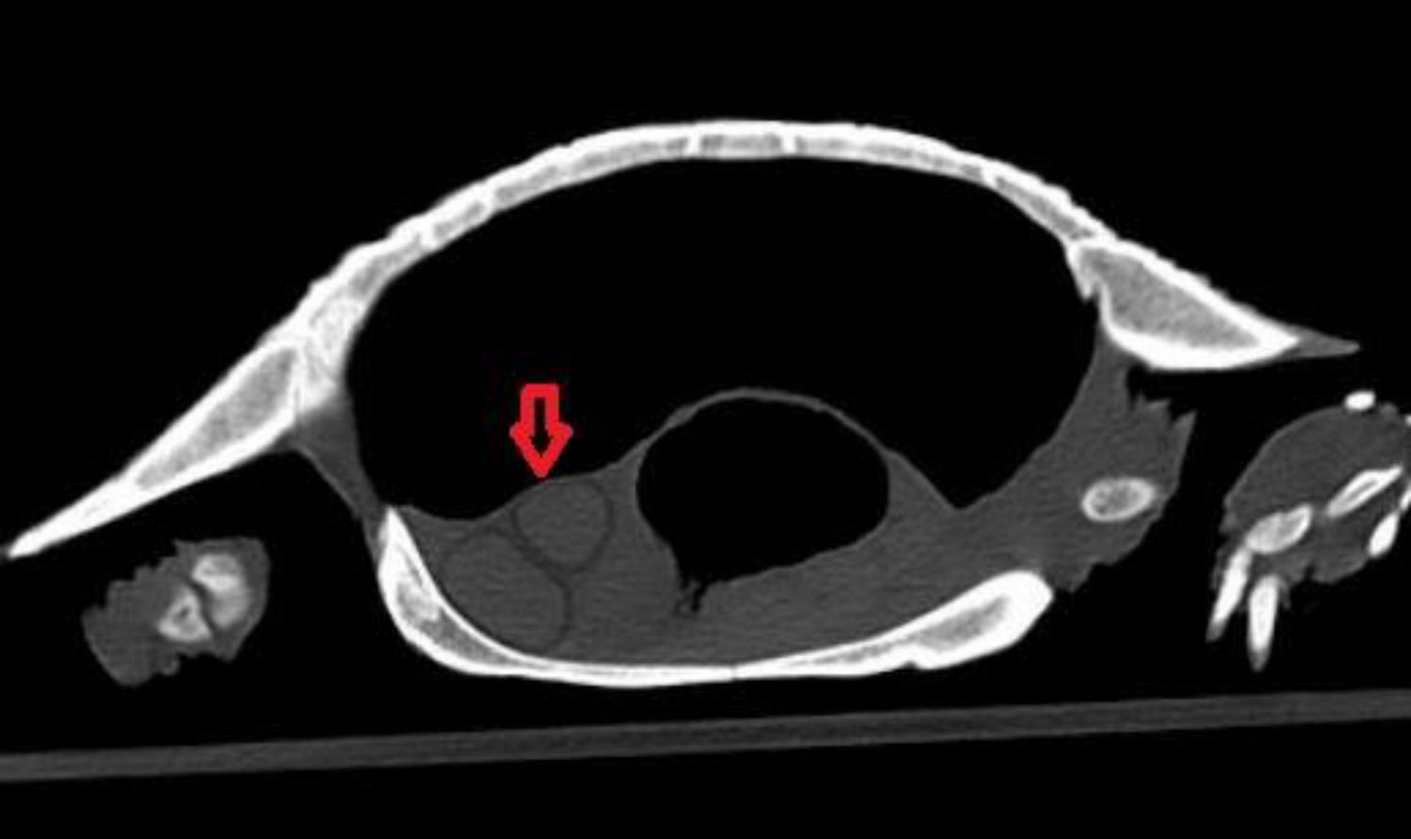
Hyperattenuated previtellogenic follicles with peripheral decreased attenuation.

**FIGURE 8:**
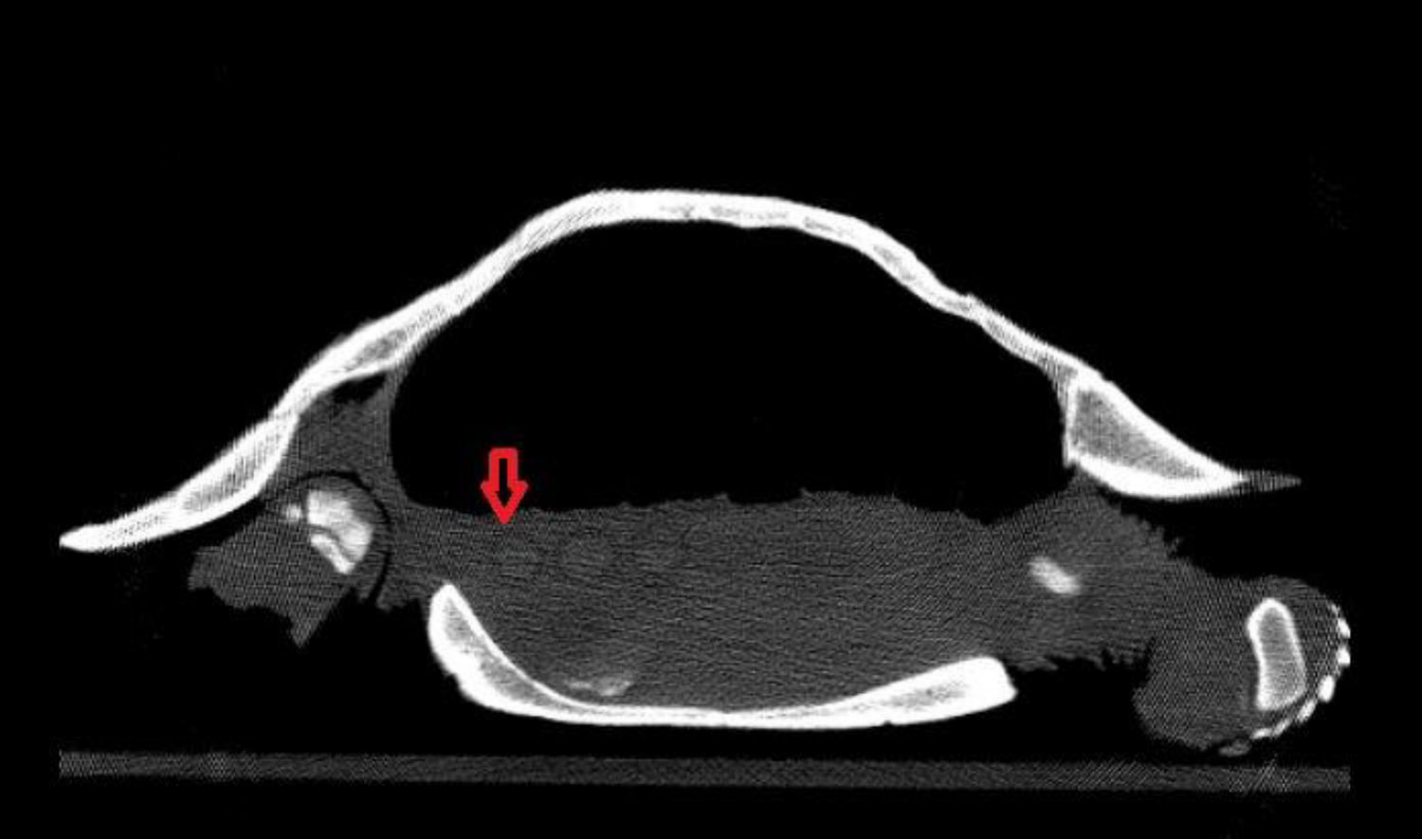
tretic follicle, the smallest and hypoattenuated follicle.

**FIGURE 9:**
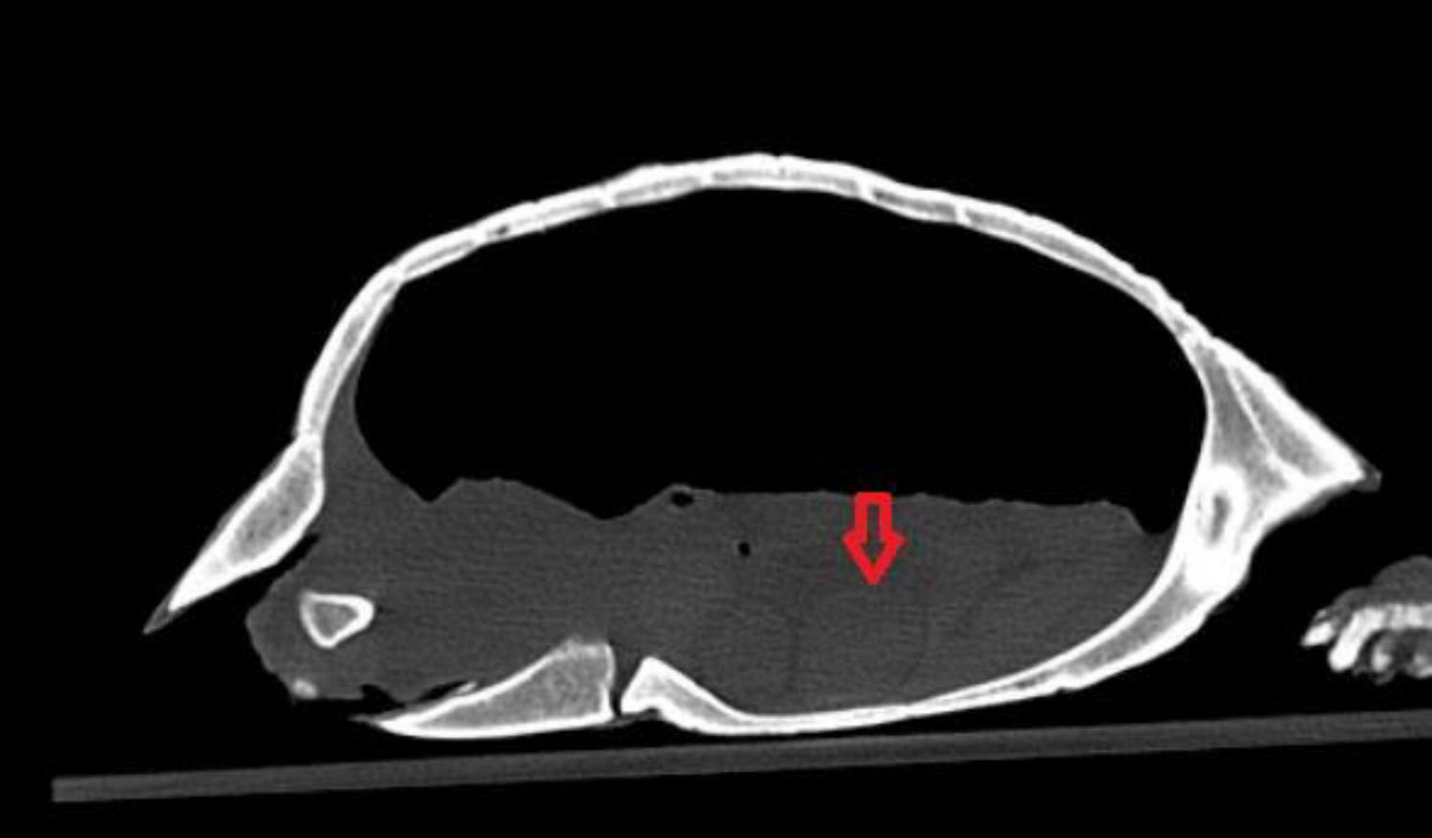
Preovulatory follicle with the most attenuation.

Different types of follicles which were detected in ultrasonography examination were also detected more easily in CT examination. Atretic follicles were the smallest and hypoattenuated follicles, the previtellogenic follicles were hyperattenuated with peripheral decreased attenuation, and preovulatory had the most attenuation compared to others (Figures 7, 8, 9).

### 3.7 Size of follicles

**FIGURE 10:**
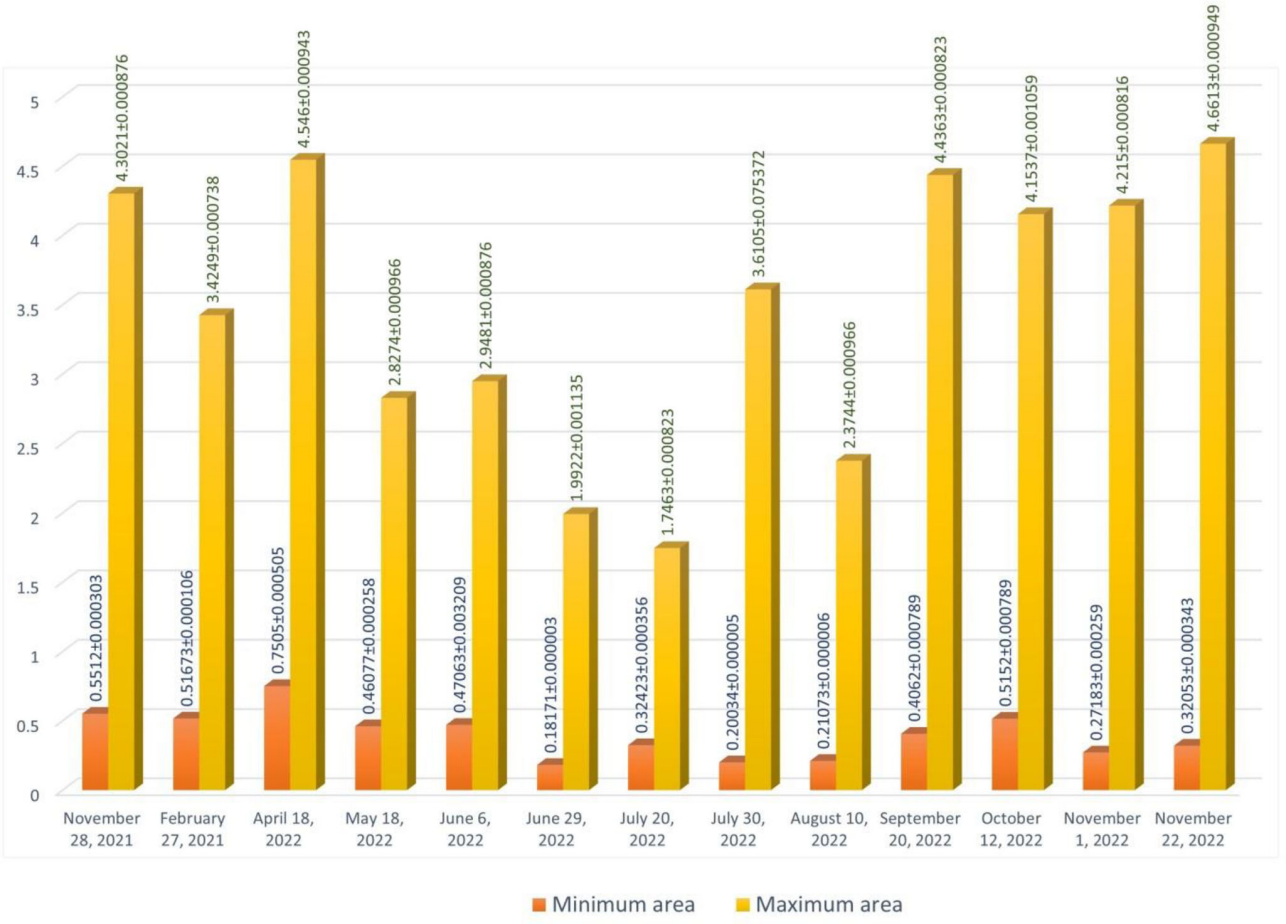
The maximum and minimum areas (mean ± SD) of follicles in computed tomographic examination of 10 tortoises (cm^2^).

Determining the size of follicles in CT was more accurate than in ultrasonography examination. Areas of follicles in different times are shown in Table 2 and column chart of Figure 10.

**TABLE 2:** The maximum and minimum areas (mean ± SD) of follicles in computed tomographic examination of 10 tortoises (cm^2^).

Minimum area (mean ± SD)	Maximum area (mean ± SD)	Date of study
0.5512 ± 0.000303	4.3021 ± 0.000876	28 November 2021
0.51673 ± 0.000106	3.4249 ± 0.000738	27 February 2021
0.7505 ± 0.000505	4.546 ± 0.000943	18 April 2022
0.46077 ± 0.000258	2.8274 ± 0.000966	18 May 2022
0.47063 ± 0.003209	2.9481 ± 0.000876	6 June 2022
0.18171 ± 0.000003	1.9922 ± 0.001135	29 June 2022
0.32423 ± 0.000356	1.7463 ± 0.000823	20 July 2022
0.20034 ± 0.000005	3.6105 ± 0.075372	30 July 2022
0.21073 ± 0.000006	2.3744 ± 0.000966	10 August 2022
0.4062 ± 0.000789	4.4363 ± 0.000823	20 September 2022
0.5152 ± 0.000789	4.1537 ± 0.001059	12 October 2022
0.27183 ± 0.000259	4.215 ± 0.000816	1 November 2022
0.32053 ± 0.000343	4.6613 ± 0.000949	22 November 2022

### 3.8 Numbers of follicles

Numbers of follicles were evaluated subjectively in 2D and 3D images of study. Considering a criterion in the first study on 28 November 2022, numbers of follicles were decreased in four proceeding studies until 6 June and then were constant in two following studies until 20 July. On 30 July, increase in number of follicles was detected with constancy until 10 August, increasing on 20 September, constant on 12 October, increasing on 1 November and finally decreased numbers of follicles on 22 November.

### 3.9 Hounsfield unit

**FIGURE 11:**
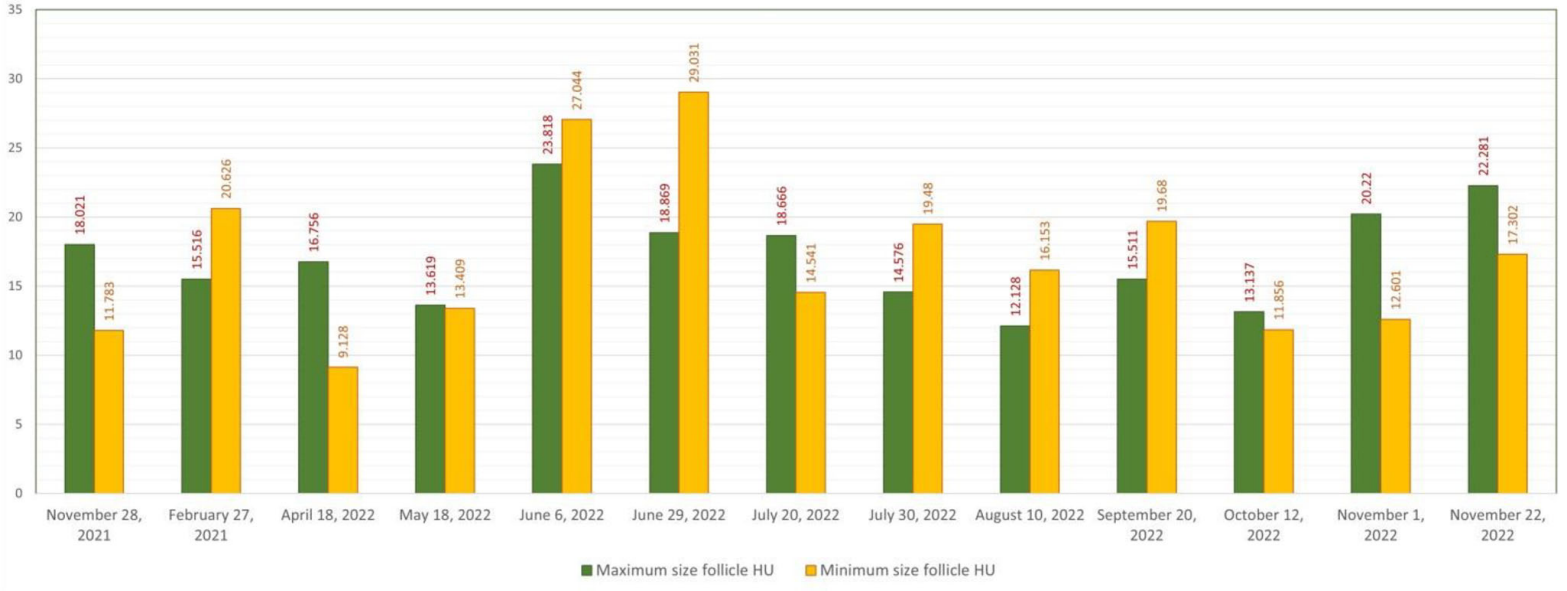
Hounsfield units of maximum and minimum size of follicles in computed tomographic examination of 10 tortoises.

Hounsfield units of the maximum and minimum size of follicles are shown in Table 3 and column chart (Figure 11).

**TABLE 3:** Hounsfield units of maximum and minimum size of follicles in computed tomographic examination of 10 tortoises.

Minimum size follicle HU	Maximum size follicle HU	Date
11.783	18.021	28 November 2021
20.626	15.516	27 February 2021
9.128	16.756	18 April 2022
13.409	13.619	18 May 2022
27.044	23.818	6 June 2022
29.031	18.869	29 June 2022
14.541	18.666	20 July 2022
19.480	14.576	30 July 2022
16.153	12.128	10 August 2022
19.680	15.511	20 September 2022
11.856	13.137	12 October 2022
12.601	20.220	1 November 2022
17.302	22.281	22 November 2022

### 3.10 Statistical analyses

Mean ± SD and the maximum/minimum size of follicles in each time of ultrasonography and computed tomographic examination were statistically analysed with Excel 2016 software.

## 4 DISCUSSION

Between two modalities which were utilized in current examination, CT had more specificity and sensitivity in detecting any types of follicles, attenuation of follicles and the numbers of detected follicles and can be completely replaced by past invasive methods such as laparoscopy and endoscopy.

According to Donini et al. (2017), the limitation of ultrasonography examination is that even though it provided a better reproductive assessment, a complete picture is often unobtainable and using multiple techniques would greatly contribute to more accurately assessing the reproductive potential of individuals in a population. In current study, this fact is completely noticeable that CT scan is the best diagnostic imaging modality to determine reproductive system of female ‘*T. graeca*’.

In some instances, complete scanning of follicles was difficult during ultrasonography because of difficulty in fanning the probe due to presence of nonflexible shell and rigidity of the rear limbs. On the other hand, radiography is the most limited method in terms of egg and follicle detection, allowing for the detection of only radio‐dense calcified eggs (Donini et al., 2017).

Urbanova and colleague in 2016 investigated the issues related to fixation and positioning of the patients; selection of suitable examination probes; and how they could be applied to a suitable body area as well as current study which dorsal recumbency positioning, hockey stick probe with 7–8 MHz frequency and prefemoral window were the optimum conditions.

The reproductive apparatus of reptiles was examined. Individual phases of gravidity in selected reptilian species were monitored; besides, the development of follicles in ovaries and the development of eggs after successful mating up to their laying (Urbanová & Halán, 2016) were evaluated.

Miguel and colleagues in 2014 observed the female reproductive cycle of captive giant tortoises (*Geochelone* spp.) by using ultrasound scanning over a 2 years period. Development of preovulatory, and atretic ovarian follicles, as well as eggs at various stages of shell deposition, were identified and measured. In their study, the same as current study, no eggs were produced by *Geochelone gigantea* during their stay in Zurich Zoo although follicles of 38–40 mm have been observed frequently in two animals (Vlachos & Tsoukala, 2014).

De Majo et al. (2016) imaged pathological features in sea turtles by using ultrasonographic examination. During the course of clinical examinations, they used 3.5 and 7.5 MHz sector transducers.

The examination was performed after placing turtles in dorsal recumbency. The right and left cervicobrachial windows allowed the visualization of the liver and gallbladder; the stomach was localized through the left prefemoral acoustic window; the intestinal loops were observed through the left and right prefemoral acoustic windows; the bladder was identified through both prefemoral acoustic windows (De Majo et al., 2016).

Costa et al. (2009) evaluated egg development of *juraras* (*Kinosternon scorpioides*) in captivity by radiography and ultrasonographic examination. The study was performed every 8 days.

In the first month, the ovarian cycle was characterized by the absence of vitellogenic follicles, atretic follicles or oviduct eggs. From October 2005 to March 2006 on, ultrasonographic scanning allowed the growing vitellogenic follicles to form. Vitellogenic follicles were observed with spherical to ovoid shapes, with a surrounding echogenic yolk, a nonechogenic albumin layer and a high echogenic shell. The oviduct eggs were identified by radiography just 180 days after beginning the experiment, when the shell became mineralized enough to impress the radiographic film. This experiment enabled the 7.5 MHz linear probe images to be obtained adequate resolution and penetration for the visualization of follicles.

Successive ultrasonographic examinations of *scorpioides* females allowed to access to initial stages of vitellogenic follicles and oviduct eggs, and radiographic examination was shown to be an easy technique to assess oviduct eggs and facilitated evaluation of egg development in *jurarás*, from 6 months on Costa et al. (2009).

Jackson and Fasal (1981) explained the exposure factors, choice of film and screens, positioning of the patient and alterations in beam direction in their study. Methods for the use of dental films and macro‐radiography are also given in their study.

Four diseases of chelonians, nutritional osteodystrophy, pneumonia, oedema and a ruptured bladder are described together with the associated radiographs and radiological interpretation (Jackson & Fasal, 1981).

Chen et al. (2011) observed the reproductive cycle of female Yellow‐Margined Box Turtle (*Cuora flavomarginata*) using radiography and ultrasonography.

Radiography was mainly used to monitor clutch size, whereas ultrasonography was applied to detect changes in the follicles throughout the year.

Ovulation occurred from March through August, and the average follicular diameter was 2.1 cm.

Follicles entered the latent period in October (at 1.5 cm), and vitellogenesis of the next reproductive cycle began in November. Using radiography, the eggshell could be detected on the ninth day after ovulation (Chen et al., 2011).

## 5 CONCLUSIONS

In current study the reproductive cycle of female *T. Graeca* in captivity is evaluated during 1 year with ultrasonography and CT scan.

It can be concluded that in captivity, the reproductive cycle does not result in the formation of eggs or calcareous shells and atretic follicles in previous year continued as before; so this should be highly considered in captivity breeding programmes.

This study also revealed that among imaging modalities, CT scan is the best modality for detecting the shape, size, type and numbers of the follicles as well as the possibility of having 3D images to further evaluate the location and shape of the follicles.

Another reason why CT is superior to other diagnostic modalities such as MRI is the lack of necessity of anaesthesia.

## AUTHOR CONTRIBUTIONS

*Writing – original draft*: Banafsheh Shateri Amiri. *Conceptualization; investigation; methodology; project administration; supervision; visualization; writing – review and editing*: Sarang Soroori, Mohammad Molazem. *Conceptualization; investigation; methodology; project administration; supervision; validation; writing – review and editing*: Amir Rostami. *Formal analysis; software*: Alireza Bahonar.

## CONFLICT OF INTEREST STATEMENT

The authors declare that they have no conflicts of interest.

## ETHICS STATEMENT

The authors confirm that the ethical policies of the journal, as noted on the journal's author guidelines page, have been adhered.

## Data Availability

No.

## Footnotes

1 Veterinarian.
